# Diabetic retinopathy further increases risk of cardiovascular disease mortality in a high-risk cohort

**DOI:** 10.1038/s41598-025-86559-x

**Published:** 2025-02-09

**Authors:** Richard Kha, Yasemin Kapucu, Mayuri Indrakumar, George Burlutsky, Aravinda Thiagalingam, Pramesh Kovoor, Paul Mitchell, Gerald Liew

**Affiliations:** 1 Centre for Vision Research, Westmead Institute for Medical Research, Westmead, NSW 2145 Australia; 2 Department of Cardiology, Westmead Hospital, Westmead, NSW Australia

**Keywords:** Coronary artery disease, Diabetic retinopathy, Mortality, Cardiovascular disease, Australian Heart Eye study, Epidemiology, Cardiology, Endocrinology

## Abstract

Diabetic retinopathy (DR) is the most common microvascular complication of diabetes and is strongly linked with systemic vascular comorbidities. This study investigated if DR predicts risk of cardiovascular disease (CVD) mortality in a high CVD risk cohort. This was a prospective cohort study of 1582 adult participants who presented to a tertiary Australian hospital for evaluation of acute coronary syndrome by coronary angiography. Participants were concurrently examined for DR from mydriatic fundus photographs which were mask-graded according to International Clinical Classification categories of no DR, mild non-proliferative DR, moderate-to-severe NPDR, and proliferative DR. Coronary artery disease was graded from coronary angiograms using the Gensini score. CVD mortality follow-up was assessed 9 years after baseline examination using linkage with the Australian National Death Index. At baseline, 355 (22.4%) participants had any DR. There were 181 (11.4%) fatal CVD events after 9-years follow-up. After controlling for age, sex, BMI, diabetes, total cholesterol, smoking status, hypertension, previous myocardial infarction and stroke, any DR was associated with 1.8-fold higher risk of CVD mortality (Hazard Ratio [HR] 1.84, 95% Confidence Interval [95% CI: 1.30–2.61]). Mild non-proliferative DR (1.85 [1.26–2.72]) and proliferative DR (5.27 [2.32–12.00]) were associated with greater CVD mortality risk. Further adjustment for coronary artery disease using Gensini scores and excluding patients without diabetes had minimal impact on the association. The increased risk of CVD mortality was significant in both men (2.25 [1.60–3.19]) and women (2.38 [1.24–4.58]) with any DR. In individuals with high CVD risk, presence of DR independently predicts increased CVD mortality. This likely reflects additional contribution of microvascular disease to CVD mortality. Individuals with DR may benefit from a comprehensive cardiovascular risk assessment, lifestyle changes, more intensive cardiovascular management and follow-up to minimise risk of death from CVD events.

**Supplementary Information:**

The online version contains supplementary material available at 10.1038/s41598-025-86559-x.

## Introduction

Diabetes mellitus has become a major public health challenge and is a significant cause of morbidity and mortality. According to the International Diabetes Federation, the number of individuals living with diabetes globally in 2019 is estimated to be 463 million^1^. This number is projected to increase to 578 million by 2030 and 700 million by 2045^1^. In 2017, diabetes was attributed to four million deaths worldwide and the global health expenditure on diabetes was around USD 727 billion^1^. Diabetes is associated with both macrovascular and microvascular complications that affect health, quality of life and lead to premature death. Macrovascular complications include accelerated cardiovascular disease, cerebrovascular events and peripheral vascular disease^2^. Microvascular complications include diabetic retinopathy (DR), neuropathy and nephropathy^2^.

Diabetic retinopathy (DR) is the most common microvascular complication of diabetes and a leading cause of vision loss in adults^3^. DR is characterised by progressive changes in the retinal vasculature including alterations in vascular permeability, capillary degeneration, capillary microaneurysms, microglia dysfunction and neovascularisation^4^. DR can be classified into non-proliferative diabetic retinopathy (NPDR) or proliferative diabetic retinopathy (PDR) based on the absence or presence of abnormal new vessels, respectively. Due to the projected increase in the global DM population, the global population with DR is expected to increase by 55.6% from 2020 to 2045^5^.

There is strong evidence linking DR with systemic vascular comorbidities including cardiovascular disease (CVD), chronic kidney disease, hypertension and cerebrovascular disease^6–11^. More importantly, the presence of DR has also been shown to be an independent risk factor for all-cause and CVD-specific mortality^12–17^. A meta-analysis of 20 observational studies found that DR resulted in a two-fold and four-fold increase in all-cause mortality in patients with type 2 and type 1 diabetes, respectively^18^. Another recent meta-analysis found that risk of all-cause mortality for type 2 diabetic patients increased by 1.38 times with NPDR and 2.32 times with PDR compared to diabetic patients without DR^19^.

However, there remain gaps in understanding the relationship between DR and mortality. While there appears a strong relationship of DR with mortality in the general population, whether the relationship still holds in participants with high baseline cardiovascular risk e.g. with existing cardiovascular co-morbidities is unclear as most studies have excluded high risk participants. Furthermore, it is unclear whether the relationship between DR and mortality is driven independently by DR or rather mediated by risk factors and complications that are commonly associated with DR such as coronary artery disease (CAD)^6,18^, as most studies utilize risk factor adjustment rather than robust measures of cardiovascular health using coronary angiograms^15,20^. Additionally, most studies to date exploring the association between DR and mortality have categorised DR into a dichotomous variable rather than analysing the different severities of DR.

Since the retina is easily viewed using non-invasive imaging techniques, being able to identify patients at a higher risk of mortality would be useful for risk stratification and patient management. This study thus aims to use robust DR grading protocols to accurately assess whether DR is a useful prognostic indicator for identifying adults with a lower chance of survival in a population with higher baseline CVD risk.

## Methods

The Australian Heart Eye Study is a population-based study that examined 1680 adults (mean age 61.06 ± 11.62 years) presenting to a large, tertiary referral hospital in the western suburbs of Sydney, New South Wales, Australia, for the evaluation of potential CAD by coronary angiography. All eligible patients provided written, informed consent to participate in the study. Ethics approval was obtained from the Western Sydney Local Health District Human Research Ethics Committee and Australian Institute of Health and Welfare (EO2018/2/435). The study was conducted in accordance with the tenets of the Declaration of Helsinki.

All eligible patients presenting for assessment of suspected CAD from June 2009 to Jan 2012 were included in the study. Participants were excluded if they did not provide written consent to data linkage for mortality or had ungradable retinal photographs.

Patients underwent a detailed medical, cardiovascular and ocular history questionnaire with trained interviewers. The medical questions included diabetes, stroke, angina, myocardial infarction, hyperlipidaemia, hypertension, surgeries, medications, drug and alcohol history. Ophthalmic questions included history and treatment for cataract, diabetic retinopathy, glaucoma, age-related macular degeneration, previous ocular disease and surgeries.

Diastolic and systolic blood pressure measurements were obtained using the OMRON digital blood pressure monitor (Model HEM-907, OMRON Healthcare, Singapore). Biochemical data including HbA1c level, fasting blood sugar, fasting cholesterol and triglyceride levels were collected from participant medical records. Hypertension was defined as previous medical diagnosis or current use of antihypertensive medications, or with systolic blood pressure > 140 mmHg or diastolic blood pressure > 90 mmHg. Diabetes was defined as current diabetic medication use, previous diagnosis of diabetes from history, HbA1c ≥ 6.5% or fasting blood glucose ≥ 7.0 mmol/L.

### Assessment of CAD

To assess coronary heart disease, patients underwent routine diagnostic cardiac angiography after 6 h of fasting, using the femoral or radial artery with a catheter of known dimension (5 Fr to 7 Fr). Coronary injections of Ultravist (Schering) were filmed in standard projections on a Siemens Bi-Plane radiographic unit (Siemens Healthcare, Germany). All angiograms were analysed by a trained cardiologist masked to the results of adjunctive investigations and retinal grading, to document the severity and extent of CAD using the Gensini scoring. This was done by first dividing the coronary artery tree into 16 segments and assigning a score according to the functional importance of the segment (5 for the left main trunk to 0.5 for the most distal segments), then multiplying by the degree of stenosis defined by the percentage reduction in luminal diameter of each narrowing (1 point for ≤ 25% narrowing, 2 points for 26–50% narrowing, 4 points for 51–75% narrowing, 8 points for 76–90% narrowing, 16 points for 91–99% narrowing, and 32 points for total occlusion). The final Gensini score is calculated by summation of the individual coronary artery segment scores, with a higher score indicating more severe CAD^21^.

### Assessment of DR

Digital retinal photographs centred on the macula and optic disc of both eyes were obtained from all participants after pharmacologic mydriasis. Seven standard Early Treatment Diabetic Retinopathy Study (ETDRS) 45° fields were taken using a digital fundus camera (Model CR-DGI, Canon Inc., Tokyo, Japan). Images were viewed at high resolution and assessed by experienced graders who were masked to participant characteristics and Gensini score. Photographic grading for the presence and severity of DR was performed according to the ETDRS grading criteria^22^, and classified according to the International Clinical Classification scale^23^.

The different stages of DR based on this classification are no DR, mild non-proliferative diabetic retinopathy (NPDR), moderate NPDR, severe NPDR, and proliferative diabetic retinopathy (PDR).

### Assessment of mortality

Demographic data from study participants were matched with the Australian National Death Index (NDI) database to confirm deaths and cause of deaths. The census cut-off point for CVD death was June 2018 (9-year follow-up). The sensitivity and specificity of Australian NDI data are estimated as 93.7% and 100% for all deaths, and 92.5% and 89.6% for cardiovascular-related deaths, respectively^24^. The cause of death in the NDI database are collected from death certificates and reported using the International Classification of Diseases (ICD) codes.

### Statistical analysis

Baseline characteristics were presented as percentage, means (SD), or median (interquartile range) by groups with or without DR. Group differences were assessed using t-tests for continuous variables and Pearson’s Chi-square test for categorical variables. Time-to-event for each participant was calculated from the date of baseline examination to the date of death or census cut-point of May 2018 (9-year follow up). Cox proportional hazards regression was used to investigate the association between DR and cardiovascular-related mortality. Multivariable models were constructed adjusting for age, sex, BMI, total cholesterol, smoking status, history of acute myocardial infarction, history of diabetes, stroke, hypertension, and Gensini score. Two-tailed p values < 0.05 were regarded as statistically significant for all tests. Kaplan-Meier survival curves for participants with and without DR were plotted for cardiovascular-related mortality. Log-rank test was used to evaluate the differences between the curves. Additional multivariable Cox regression models stratified by age and sex were also constructed. Finally, a sensitivity analysis was performed which included participants with known diabetes only. All statistical analyses were conducted using SAS version 9.2 (SAS Institute Inc, Cary, NC).

## Results

Out of 1680 participants, 1582 (94.2%) had gradable fundus photographs and were included in this study. Of these participants, 355 participants were identified as having any DR (22.4%), of whom 210 (13.3%) had mild NPDR, 124 (7.8%) had moderate-to-severe NPDR and 21 (1.3%) had proliferative DR. 633 participants (40.0%) were classified as having diabetes, of which 111 participants were newly diagnosed with diabetes. The cohort consisted of more males (75.9%) than females (24.2%). Table 1 outlines the demographic and clinical characteristics of participants with and without DR. Participants identified as having DR were more likely to be older, with higher Gensini score, have previous history of diabetes, hypertension, stroke and higher BMI compared to participants without DR. Additionally, the group with DR had lower total cholesterol, and were less likely to smoke.

**Table 1:** Baseline characteristics of the study population, by presence and absence of diabetic retinopathy.

Characteristic	Any Diabetic Retinopathy (*N* = 355)	No Diabetic Retinopathy (*N* = 1227)	*P*-value
Age, yrs, mean (SD)	62.63 (11.23)	60.87 (11.72)	0.01
Sex, n (%)			0.97
Male	269 (75.8)	931 (75.9)	
Female	86 (24.2)	296 (24.1)	
Smoking status, n (%)			0.007
Current smoker	75 (21.2)	358 (29.3)	
Ex-smoker	139 (39.3)	404 (33.0)	
Never smoked	140 (39.5)	461 (37.7)	
Prior history of diabetes, n (%)	244 (68.9)	278 (22.7)	< 0.0001
Hypertension, n (%)	285 (80.7)	756 (61.8)	< 0.0001
Total cholesterol, mmol/L, mean (SD)	4.34 (1.20)	4.67 (1.14)	0.0001
BMI, kg/m^2^, mean (SD)	30.29 (6.21)	29.29 (5.42)	0.02
Gensini score, units, mean (SD)	42.50 (41.42)	36.00 (37.19)	0.02
History of acute myocardial infarction, n (%)	101 (28.9)	321 (26.4)	0.35
History of angina, n (%)	198 (55.8)	707 (57.8)	0.51
History of stroke, n (%)	21 (6.0)	33 (2.7)	0.003

BMI, body mass index; SD, standard deviation.

Table 2 shows Cox proportional hazard regression models for DR and CVD mortality. Over 9 years of follow-up, there were 181 (11.5%) deaths due to CVD. In model 1, adjustment is performed for age, sex, BMI, total cholesterol, smoking status, history of diabetes, myocardial infarction, stroke and hypertension, and in model 2 additionally for Gensini score. Adjustment for potential confounders in model 1 showed that compared to participants without DR, having any DR (Hazard Ratio, HR = 1.84, 95% Confidence Intervals: 1.30–2.61), mild NPDR (HR = 1.85, 95% CI: 1.26–2.72) and PDR (HR = 5.27, 95% CI: 2.32–12.00) were significantly associated with CVD mortality. Further adjustment for Gensini score in model 2 had minimal influence on the hazard ratios of CVD mortality for any DR (HR = 1.80, 95% CI: 1.21–2.70), mild NPDR (HR = 1.70, 95% CI: 1.08–2.68) and PDR (HR = 4.67, 95% CI: 1.60–13.62) as they remained similar to model 1 and were statistically significant.

**Table 2:** Hazard ratios for cardiovascular disease-related mortality events among study participants grouped by diabetic retinopathy status.

Diabetic retinopathy severity	*N* (%)	Age-sex adjusted HR (95% CI)	*p*-value	Multivariable adjusted HR model 1 (95% CI)*	*p*-value	Multivariable adjusted HR model 2 (95% CI)**	*p*-value
No DR	1227/1582 (77.6)	Reference		Reference		Reference	
Any DR	355/1582 (22.4)	2.39 (1.77–3.22)	< 0.0001	1.84 (1.30–2.61)	< 0.0001	1.80 (1.21–2.70)	0.004
Mild NPDR	210/1582 (13.3)	2.20 (1.55–3.14)	< 0.0001	1.85 (1.26–2.72)	0.002	1.70 (1.08–2.68)	0.02
Moderate to severe NPDR	124/1582 (7.8)	2.30 (1.44–3.67)	0.0005	1.50 (0.86–2.60)	0.16	1.82 (0.96–3.44)	0.07
Proliferative DR	21/1582 (1.3)	6.67 (3.09–14.38)	< 0.0001	5.27 (2.32–12.00)	< 0.0001	4.67 (1.60–13.62)	0.005

CI,  confidence intervals; HR, hazard ratio; DR , diabetic retinopathy; NPDR , non-proliferative diabetic retinopathy.

*Adjusted for age, sex, BMI, total cholesterol, smoking status, history of diabetes, acute myocardial infarction, history of stroke and hypertension.

**Adjusted for age, sex, BMI, total cholesterol, smoking status, history of diabetes, acute myocardial infarction, history of stroke, hypertension, and Gensini score.

Supplementary Table S1 and Supplementary Table S2 display the results of the subgroup analyses stratified by age and sex, respectively. In subgroup analyses stratified by age, only PDR was significantly associated with an increased likelihood of CVD mortality in patients younger than 65 years old (HR = 5.55, 95% CI: 1.40–22.01). Mild NPDR (HR = 1.76, 95% CI: 0.83–3.74) and moderate-to-severe NPDR (HR = 1.34, 95% CI: 0.48–3.72) were not associated with CVD mortality. In patients who were 65 years or older, any DR (HR = 2.06, 95% CI: 1.35–3.15), mild NPDR (HR = 2.16, 95% CI: 1.36–3.44) and PDR (HR = 7.33, 95% CI: 2.53–21.25) were significantly associated with greater CVD mortality. In subgroup analyses stratified by sex, the association of any DR with CVD mortality was similar in males (HR = 2.02, 95% CI: 1.40–2.92) and females (HR = 2.38, 95% CI: 1.24–4.58).

Finally, when the analysis was restricted to only participants with known i.e. previous diagnosis of diabetes, the findings remained similar with multivariate adjusted model 2 retaining significant association of DR with CVD mortality for any DR (HR = 1.70, 95% CI: 1.09–2.65), mild NPDR (HR = 1.79, 95% CI: 1.07–3.01) and PDR (HR = 5.28, 95% CI: 2.24–12.46), whereas moderate-to-severe NPDR remained non-significant (HR = 1.26, 95% CI: 0.68–2.34). These results are shown in Supplementary Table S3.

**Fig. 1:**
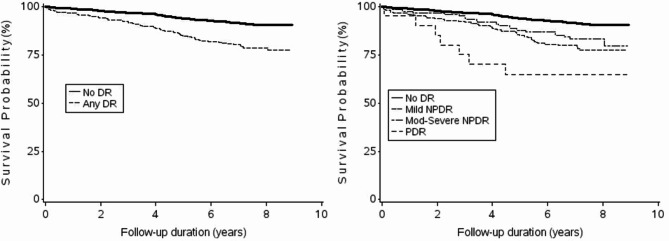
Kaplan-Meier curves showing cardiovascular disease mortality-free survival in participants with and without diabetic retinopathy.

Figure 1 shows the adjusted Kaplan-Meier curves for CVD mortality based on the presence and severity of DR. The results indicated that participants with DR had significantly lower survival probabilities during the 9-year follow-up (χ^2^ = 40.35, *p* < 0.0001) compared to participants without DR. The curve showing severity of DR highlights that as severity of DR increased, the rate of survival progressively decreased.

## Discussion

DR is known to increase mortality in the general population, and this prospective cohort found that the relationship also applies in high cardiovascular risk populations. The study showed that any DR was significantly associated with subsequent CVD-related mortality in diabetic patients, and this risk increased exponentially with PDR. After adjustment for cardiovascular risk factors and macrovascular CAD severity using the Gensini score, our results showed that compared to patients without DR, the risk of CVD-related mortality was almost two times greater in those with any DR and five times greater in those with PDR, which is the most severe form of DR. This suggests that DR is a strong risk factor for CVD-related mortality, independent of cardiovascular risk factors, baseline high CVD risk, as well as existing macrovascular disease. The effect of DR on CVD mortality was similar in males and females, however the effect was considerably greater in patients 65 years and older, compared to patients who were younger than 65 years.

Our study builds on the findings of other studies examining the relationship between DR and CVD mortality risk. These include the SIDIAP cohort study (HR = 1.22, 95% CI: 1.14–1.31 for any DR)^25^, SEED study (HR = 1.74, 95% CI: 1.27–2.40 for any DR; HR = 1.95; 95% CI: 1.20–3.19 for moderate NPDR; HR = 3.41, 95% CI: 2.11–5.50 for severe NPDR and PDR)^26^, 2005–2008 NHANES study (HR = 1.87, 95% CI: 1.29–2.71 for any DR)^27^ and AGES-RS study (HR = 1.57, 95% CI: 1.20–2.06 for any DR)^16^, which reported that the presence and severity of DR has an independent effect on increasing risk of CVD mortality. In a meta-analysis of 11,239 diabetic patients, the risk of CVD mortality was 1.83 times and 2.26 times higher in those with any DR and severe DR, respectively^28^. A meta-analysis of 20 observational studies by Kramer et al. found that DR increased the likelihood for CVD events by 2.34 compared to patients without DR^18^. An umbrella review in 2020 suggested a greater chance of CVD mortality for any DR (HR = 1.83, 95% CI: 1.42–2.36) and severe DR (HR = 2.26, 95% CI: 1.31–3.91)^29^. Compared to previous studies, the hazard ratios for CVD mortality demonstrated in our study was significant for any DR, but higher for patients with more advanced retinopathy such as PDR. A possible explanation for this could be the fact that our study cohort had a higher baseline risk of CVD since it consisted of patients who were assessed for suspected CAD. However, it could also be due to differences in methodology compared to other studies, as we used precise retinopathy imaging and grading, including measuring macrovascular CAD directly using coronary angiography and the Gensini score, which to our knowledge has not been performed previously.

There are other studies suggesting that the association between DR and CVD mortality is primarily due to shared risk factors and does not persist after confounder adjustment. These studies include findings from the EURODIAB study^17^, Hoorn study^30^, and 1988–1994 NHANES study^31^ which did not find a statistically significant association between DR and CVD mortality. However, the EURODIAB study had missing fundus photographs (31%) and morbidity data (14%) which could have resulted in underestimation of association between DR and mortality. The NHANES study used photographs from one randomly selected eye which may have underestimated the prevalence of retinopathy. Additionally, the Hoorn study had a small sample size (*n* = 631) which may have been underpowered to detect statistically significant difference. Our study addressed these limitations and provided strong evidence that DR is a strong, graded and independent risk factor for CVD mortality.

The pathophysiology behind the association of DR and mortality is unclear. It appears independent of shared risk factors, and we speculate may represent the additional contribution of microvascular disease to CVD mortality. DR is associated with coronary circulation abnormalities, including reduced coronary flow reserve, myocardial perfusion and increased coronary artery stenosis^32,33^. These processes include components of inflammation, advanced glycated end products, endothelial dysfunction, apoptosis, neovascularization and oxidative stress, and these can lead to both microvascular and macrovascular disease^34–37^. Adjustment for macrovascular disease via the Gensini score did not weaken the association, suggesting the association may reflect the existence of microvascular disease in the cardiac circulation, similar to how DR reflects microvascular disease in the retinal circulation. Another potential explanation is that microvascular disease has a direct influence on macrovascular disease. DR alters the autonomic nervous system function, resulting in progression of cardiac autonomic neuropathy^38^. This leads to exercise intolerance, silent myocardial infarctions, orthostatic hypotension, cardiac rhythm dysregulation and is strongly linked with greater rates of cardiac events and CVD mortality^39,40^.

Key strengths of our study include accurate assessment of study variables, such as grading of retinopathy from seven 45° ETDRS field fundus photographs, grading severity of coronary heart disease using the coronary angiography derived Gensini score and ascertainment of CVD mortality data from the validated NDI.

Several limitations exist in our study. First, the study cohort consisted of patients who presented for evaluation of potential CAD. Since this is a selected population, the findings may not be generalisable to low risk populations, and may be of more relevance to patients presenting to cardiology or diabetes clinics. Secondly, our main analyses included participants with and without diabetes to preserve study power. In supplementary analyses where we excluded participants with a history of diabetes, the results remained broadly similar, with similar HRs but mild NPDR becoming borderline non-significant due to loss of statistical power. Finally, we were unable adjust for HbA1c or diabetes duration due to the lack of complete data for these variables in this study. Although these have been adjusted for in some previous studies, those studies have found that they had little impact on the association between DR and CHD^11,15^. Indeed, in our univariate analyses, these variables were not associated with CVD mortality, and hence not included in the final multivariate models. Thus, these variables are unlikely to change the association between DR and CVD observed in our study.

## Conclusions

We report that the presence and severity of DR is significantly associated with increased risk of CVD mortality, even in a cohort at high baseline CVD risk. The association was independent of cardiovascular risk factors and macrovascular CAD severity, supporting the role of DR in reflecting the additional contribution of microvascular disease to CVD mortality. The relationship between DR and CVD mortality was similar between sex but was stronger in patients who were 65 years or older compared to those who were younger than 65 years. Individuals with DR, especially older adults, may benefit from a comprehensive cardiovascular risk assessment, lifestyle changes, more intensive cardiovascular management and follow-up to minimise risk of death from CVD events.

## Electronic supplementary material

Below is the link to the electronic supplementary material.

Supplementary Material 1

## Data Availability

The datasets generated during and/or analysed in the current study are available from the corresponding author upon reasonable request.

## Abbreviations

BMI: Body mass index
CAD: Coronary artery disease
CI: Confidence interval
CVD: Cardiovascular disease
DR: Diabetic retinopathy
ETDRS: Early Treatment Diabetic Retinopathy Study
HR: Hazard ratio
NDI: National Death Index
NPDR: Non-proliferative diabetic retinopathy
PDR: Proliferative diabetic retinopathy
SD: Standard deviation
