# Supplementary material for: Diabetic retinopathy further increases risk of cardiovascular disease mortality in a high-risk cohort
Source: Sci Rep. 2025 Feb 9;15:4811. doi: 10.1038/s41598-025-86559-x (PMC11808117; doi:10.1038/s41598-025-86559-x)
Supplement: Supplementary file 1 — Supplementary Material 1 [file 41598_2025_86559_MOESM1_ESM.docx]

**Supplementary Table S1.** Hazard ratios for diabetic retinopathy and cardiovascular disease-related mortality events among study participants stratified by age.

| **Diabetic retinopathy severity** | **Multivariable adjusted HR model** | **p-value** |
| --- | --- | --- |
| *Age <65 years* | | |
| No DR | Reference | Reference |
| Any DR | 1.74 (0.89 – 3.40) | 0.11 |
| Mild NPDR | 1.76 (0.83 – 3.74) | 0.14 |
| Moderate to severe NPDR | 1.34 (0.48 – 3.72) | 0.58 |
| Proliferative DR | 5.55 (1.40 – 22.01) | 0.01 |
| *Age ≥ 65 years* |  |  |
| No DR | Reference | Reference |
| Any DR | 2.06 (1.35 – 3.15) | 0.0008 |
| Mild NPDR | 2.16 (1.36 – 3.44) | 0.001 |
| Moderate to severe NPDR | 1.53 (0.80 – 2.94) | 0.20 |
| Proliferative DR | 7.33 (2.53 – 21.25) | 0.0002 |

*Adjusted for age, sex, BMI, total cholesterol, smoking status, history of diabetes, acute myocardial infarction, history of stroke and hypertension.

**Supplementary Table S2.** Hazard ratios for diabetic retinopathy and cardiovascular disease-related mortality events among study participants stratified by sex.

| **Diabetic retinopathy severity** | **Multivariable adjusted HR model** | **p-value** |
| --- | --- | --- |
| *Male* | | |
| No DR | Reference | Reference |
| Any DR | 2.02 (1.40 – 2.92) | 0.0002 |
| Mild NPDR | 2.01 (1.32 – 3.06) | 0.001 |
| Moderate to severe NPDR | 1.90 (1.06 – 3.39) | 0.03 |
| Proliferative DR | 3.64 (1.12 – 11.82) | 0.03 |
| *Female* |  |  |
| No DR | Reference | Reference |
| Any DR | 2.38 (1.24 – 4.58) | 0.009 |
| Mild NPDR | 2.21 (0.97 – 5.03) | 0.06 |
| Moderate to severe NPDR | 1.45 (0.47 – 4.44) | 0.52 |
| Proliferative DR | 17.98 (5.44 – 59.41) | <0.0001 |

*Adjusted for age, sex, BMI, total cholesterol, smoking status, history of diabetes, acute myocardial infarction, history of stroke and hypertension.

**Supplementary Table S3.** Sensitivity analysis for cardiovascular disease-related mortality events in participants with known diabetes only, grouped by diabetic retinopathy status

| **Diabetic retinopathy severity** | **Multivariable adjusted HR model*** | **p-value** |
| --- | --- | --- |
| No DR | Reference | Reference |
| Any DR | 1.70 (1.09 – 2.65) | 0.02 |
| Mild NPDR | 1.79 (1.07 – 3.01) | 0.03 |
| Moderate to severe NPDR | 1.26 (0.68 – 2.34) | 0.46 |
| Proliferative DR | 5.28 (2.24 – 12.46) | 0.0001 |

*Adjusted for age, sex, BMI, total cholesterol, smoking status, history of diabetes, acute myocardial infarction, history of stroke and hypertension.
